# Transcriptome Profiling of m^6^A mRNA Modification in Bovine Mammary Epithelial Cells Treated with *Escherichia coli*

**DOI:** 10.3390/ijms22126254

**Published:** 2021-06-10

**Authors:** Ting Li, Changjie Lin, Yifan Zhu, Haojun Xu, Yiya Yin, Chaohao Wang, Xin Tang, Tongxing Song, Aizhen Guo, Yingyu Chen, Changmin Hu

**Affiliations:** 1 Department of Clinical Veterinary Medicine, Faculty of Veterinary Medicine, Huazhong Agricultural University, Wuhan 430070, China; tasjlt@webmail.hzau.edu.cn (T.L.); 875155626@webmail.hzau.edu.cn (C.L.); a845911819@163.com (H.X.); yyyfighting0118@163.com (Y.Y.); 15310426171@163.com (C.W.); tangxin1995@webmail.hzau.edu.cn (X.T.); 2 State Key Laboratory of Agricultural Microbiology, Huazhong Agricultural University, Wuhan 430070, China; avander1@163.com (Y.Z.); aizhen@mail.hzau.edu.cn (A.G.); chenyingyu@mail.hzau.edu.cn (Y.C.); 3 Department of Preventive Veterinary Medicine, Faculty of Veterinary Medicine, Huazhong Agricultural University, Wuhan 430070, China; 4 Department of Animal Nutrition and Feed Science College of Animal Science and Technology, Huazhong Agricultural University, Wuhan 430070, China; songtongxing@mail.hzau.edu.cn

**Keywords:** mastitis, MeRIP-seq, *E. coli*, m^6^A modification, *N*^6^-methyladenosine

## Abstract

Mastitis is a common disease in dairy cows that is mostly caused by *E. coli*, and it brings massive losses to the dairy industry. *N*^6^-Methyladenosine (m^6^A), a methylation at the *N*^6^ position of RNA adenine, is a type of modification strongly associated with many diseases. However, the role of m^6^A in mastitis has not been investigated. In this study, we used MeRIP-seq to sequence the RNA of bovine mammary epithelial cells treated with inactivated *E. coli* for 24 h. In this in vitro infection model, there were 16,691 m^6^A peaks within 7066 mRNA transcripts in the Con group and 10,029 peaks within 4891 transcripts in the *E. coli* group. Compared with the Con group, 474 mRNAs were hypermethylated and 2101 mRNAs were hypomethylated in the *E. coli* group. Biological function analyses revealed differential m^6^A-modified genes mainly enriched in the MAPK, NF-κB, and TGF-β signaling pathways. In order to explore the relationship between m^6^A and mRNA expression, combined MeRIP-seq and mRNA-seq analyses revealed 212 genes with concomitant changes in the mRNA expression and m^6^A modification. This study is the first to present a map of RNA m^6^A modification in mastitis treated with *E. coli*, providing a basis for future research.

## 1. Introduction

Mastitis is one of the most severe diseases in dairy farming, and its diagnosis and treatment present many challenges. Common measures to prevent mastitis include antibiotic treatments, the discarding of milk, and the elimination of sick cows, all of which cause serious losses [1]. Over the past decades, studies of mastitis have mainly focused on the reaction between the pathogen and the host [2]. The innate immune system of the mammary gland has been shown to minimize mastitis caused by pathogenic microorganisms, thereby cooperating with the acquired immune system. Mammary epithelial cells (MECs), as the first barrier of nonspecific immunity of mastitis, play a crucial role in mastitis following infection by pathogenic microorganisms. These cells secrete a variety of inflammatory factors and chemokines [3] that regulate cell apoptosis, the Toll-like receptor (TLR) pathway, the mitogen-activated protein kinase (MAPK) pathway, and other signaling pathways [4]. Mastitis-associated *Escherichia coli (E. coli*) [5] could result in an acute course of mastitis with severe clinical symptoms [6,7]. Lipopolysaccharides (LPS), a component of *E. coli*, can activate TLR signal transduction and cause a series of reaction cascades [8,9,10,11]. It has also been reported that *E. coli* can induce apoptosis in mastitis [12]. These factors eventually lead to inflammation and tissue damage. Although many studies have been conducted on mastitis, knowledge of its diagnosis and treatment remains insufficient. Therefore, new research on pathogen-specific mastitis may provide new directions for diagnosis and treatment.

*N*^6^-Methyladenosine (m^6^A) is the most common RNA modification in eukaryotes, and it involves methylation at the *N*^6^ position of RNA adenine [13,14]. In the 1970s, m^6^A modification was detected in eukaryotic mRNA and lncRNA [15], and subsequent studies found that about 1–2% of mRNAs contain m^6^A modification [16]. Studies have demonstrated that m^6^A modification is vital to splicing and editing of mRNA, degradation of polyadenylation, and other RNA processing events [17]. Furthermore, m^6^A modification may promote mRNA nuclear export and translation initiation, and it maintains the structural stability of mRNA with poly A binding proteins. m^6^A modification is mainly involved in three enzymes: “writers”, methylation transferases responsible for the methylation modification of RNA, such as the METTLE family and WTAP; “erasers”, demethylation enzymes, including FTO and ALKBH5, which clear the methylation modification of RNA; “readers”, methylation-reading proteins, such as YTH family protein and elF3 [18,19,20,21,22]. m^6^A modification is involved in many pathophysiological processes in mammals [23]. Chen et al. found that m^6^A modification of circular RNA inhibited innate immunity [24]. Yu et al. reported that m^6^A reader protein YTHDF was closely correlated with inflammation induced by LPS via the expression of inflammatory genes such as nuclear factor kappa B (NF-κB) and MAPK [25]. Zhu and Lu reported that the “eraser” ALKBH5 could regulate the cell apoptosis induced by LPS [26]. However, the nature of the relationship between mastitis and m^6^A modification is still unclear.

In this study, we determined potential m^6^A modification in the inflammation of bovine mammary epithelial cells treated with inactivated *E. coli* using high-throughput sequencing (MeRIP-seq). Differential m^6^A-modified transcripts were analyzed in *E. coli*-induced MAC-T. Furthermore, we performed biological function analysis of the differential m^6^A modification and clustered signaling pathways of differentially methylated mRNAs. We also investigated the relationship between m^6^A modification and mRNA transcription. These findings lay a foundation for further exploration of m^6^A RNA modification in mastitis treated with *E. coli*.

## 2. Results

### 2.1. Establishment of Mastitis Model In Vitro

**Figure 1:**
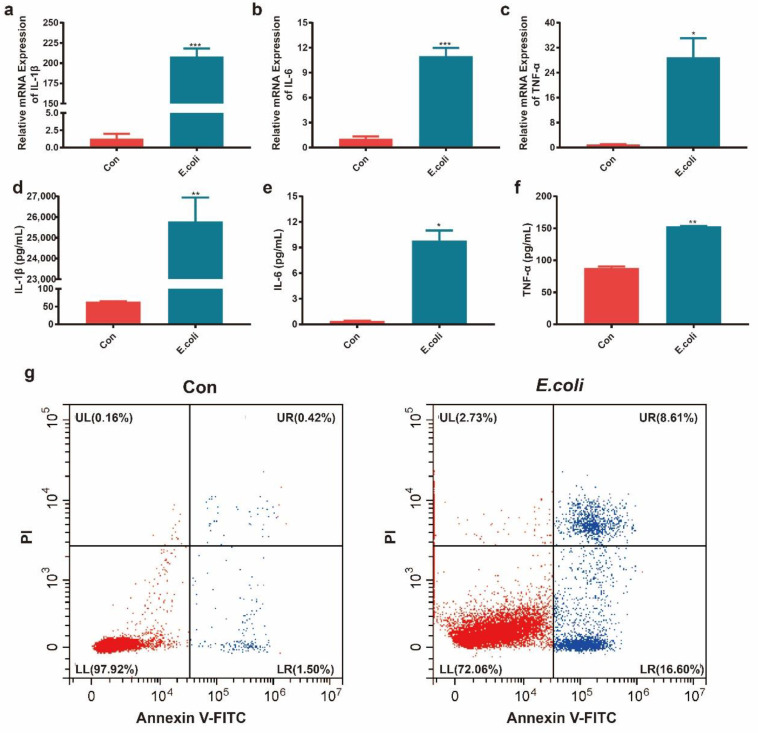
Inflammatory factor expression and apoptotic cell rate in the mastitis model in vitro. (**a**–**c**) Relative mRNA levels of IL-1β, IL-6, and TNF-α measured using real-time PCR in the Con and *E. coli* groups. (**d**–**f**) Concentrations of IL-1β, IL-6, and TNF-α measured using ELISA in the Con and *E. coli* groups. (**g**) The apoptotic cell rate in the Con and *E. coli* groups according to flow cytometry. Data are expressed as the mean ± standard deviation (SD) and analyzed using one-way analysis of variance; *, **, and *** represent *p* < 0.05, *p* < 0.01, and *p* < 0.001, respectively.

In this study, MAC-T cells were treated with heat-inactivated *E. coli* with an MOI of 10:1 for 24 h. We detected the mRNA and protein expression of IL-1β, IL-6, and TNF-α by RT-qPCR (Figure 1a–c) and ELISA (Figure 1d–f). The results show that the expression levels of proinflammatory factors IL-1β, IL-6, and TNF-α were significantly increased in the MAC-T cells treated with inactivated *E. coli* (*p* < 0.05). Meanwhile, we used flow cytometry to detect the apoptosis of MAC-T cells induced by inactivated *E. coli*. The results show that the percentage of apoptotic cells in the control group was 1.92% and that in the *E. coli* group was 25.21% (Figure 1g), suggesting that inactivated *E. coli* could cause MAC-T cell apoptosis.

### 2.2. Profile of the m^6^A Modification in MAC-T Cells Treated with E. coli

**Figure 2:**
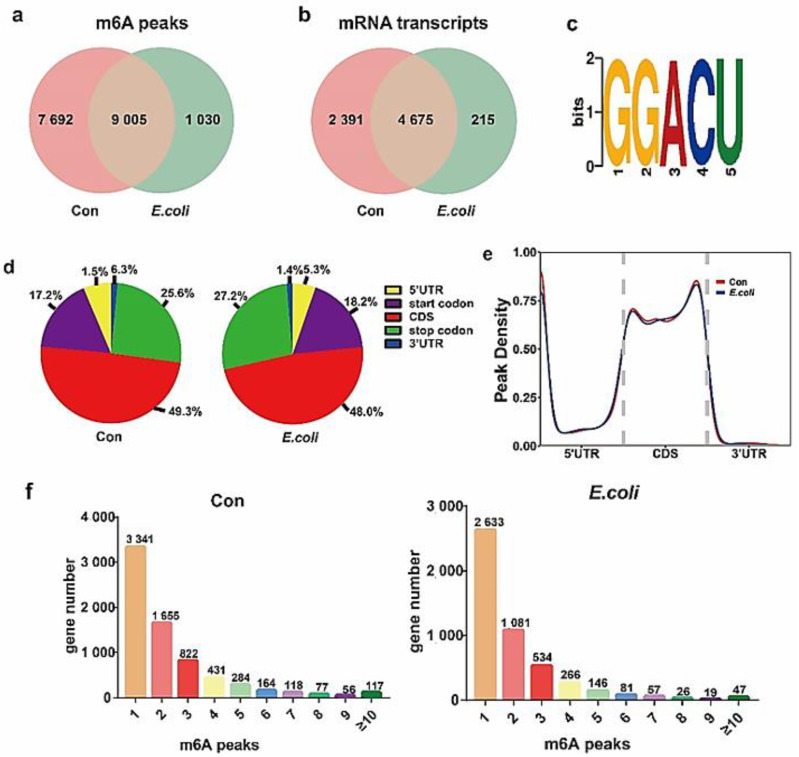
Overview of m^6^A methylation map in Con and *E. coli* groups. (**a**) Venn diagram showing the specific and common peaks between two groups. (**b**) Venn diagram displaying the specific and common mRNA transcripts between the Con and *E. coli* groups. (**c**) RRACH sequence motif enrichment of the m^6^A peaks in the Con and *E. coli* groups. (**d**) Pie charts demonstrating m^6^A peak distribution in the gene structures of mRNAs. (**e**) Metagene plots displaying the regions of m^6^A peaks identified across the transcripts in the Con and *E. coli* groups. (**f**) The number of m^6^A peaks per gene in the Con and *E. coli* groups.

In order to obtain a map of m^6^A modification in MAC-T cells treated with *E. coli*, we performed a transcriptome profiling of m^6^A modification analysis using meRIP-seq. Comparing the clean reads to the reference genome, there were 16,691 m^6^A peaks within 7066 mRNA transcripts in the Con group and 10,029 peaks within 4891 mRNA transcripts in the *E. coli* group (Figure S1a,b, Supplementary Materials). A total of 9005 m^6^A modification peaks and 4675 mRNA transcripts existed in the two groups, whereby 7692 peaks and 2391 mRNA transcripts existed in the Con group and 1030 peaks and 215 mRNA transcripts existed in the *E. coli* group (Figure 2a,b). The most enriched motif sequence [27,28] m^6^A peak in the Con and *E. coli* groups was GGACU (Figure 2c).

An analysis of the specific positions of peaks on modified genes revealed that methylation in the CDS region was significantly greater than in other regions in the Con and *E. coli* groups (Figure 2d), consistent with the results reported in previous studies [29]. Furthermore, the distribution density of methylation peaks in the Con and *E. coli* groups was highly similar (Figure 2e). Studies have confirmed that there is sometimes more than one methylation peak of mRNA, but one peak is usually the most common. In this study, a count of the methylation peaks of mRNAs revealed that the number of mRNAs with one methylation peak was the largest, although genes with multiple methylation peaks were not uncommon (Figure 2f).

### 2.3. Differential m^6^A Modification between the E. coli Group and Control Group

**Figure 3:**
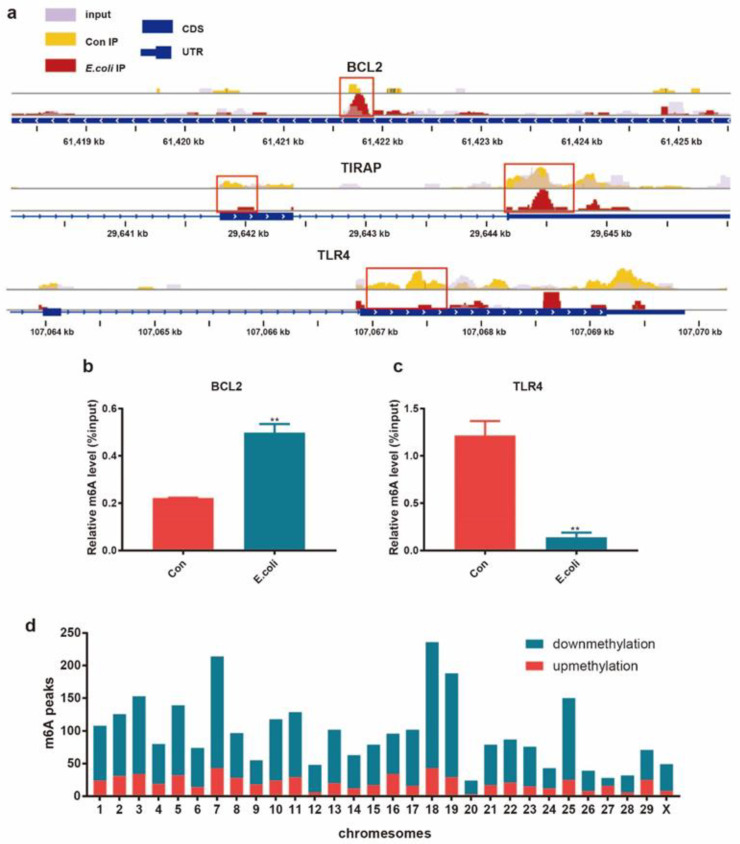
Distribution of significantly differential peaks between the Con and *E. coli* groups. (**a**) Data visualization analysis of differential m^6^A peaks in selected mRNAs (BCL2, TIRAP, and TLR4) in the *E. coli* group compared with the Con group. MeRIP-qPCR verified the differential m^6^A modification in BCL2 (**b**) and TLR4 (**c**). (**d**) Distribution of differential m^6^A sites on bovine chromosomes.

In order to further study the role of m^6^A modification in dairy bovine mastitis, we analyzed the differences between the m^6^A peaks of RNA in the *E. coli* and Con groups. Compared with the Con group, there were 2904 significantly differential m^6^A peaks within 2101 mRNAs in the *E. coli* group (Table S2, Supplementary Materials), of which 644 hypermethylated peaks were within 474 mRNAs such as BCL2 (Figure 3a) and 2260 hypomethylated peaks were within 1627 mRNAs, such as TIRAP and TLR4 (Figure 3a) (*p* < 0.00001, fold change >2.0). Table 1 shows the top 10 hypermethylated or hypomethylated genes in *E. coli* groups compared with the Con group. This study also demonstrated that BCL2 was m^6^A hypermethylated (Figure 3b), while TLR4 was m^6^A hypomethylated (Figure 3c) in *E. coli*-induced MAC-T cells. Additionally, compared with the Con group, several differential methylation sites in the *E. coli* group were located on chromosomes 7, 18, 19, and 25 (Figure 3d).

### 2.4. Differential m^6^A Modifications Participate in Important Biological Pathways

**Figure 4:**
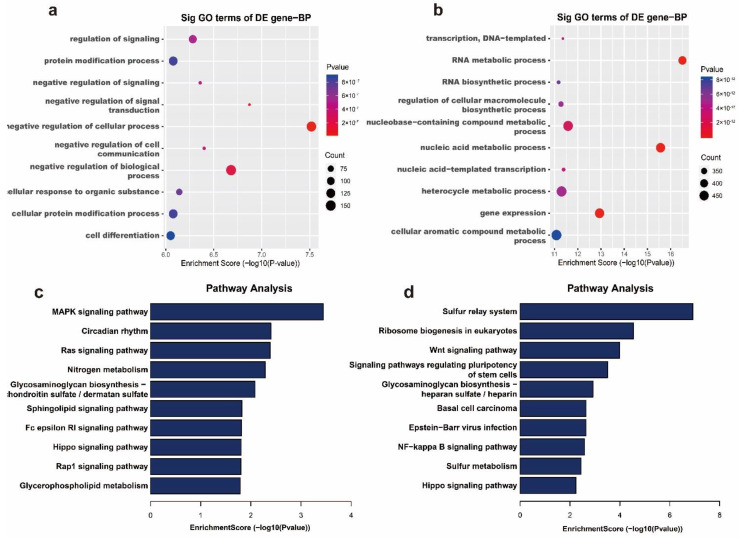
Biological function analysis of differential m^6^A modification according to GO biological processes (BPs) and KEGG pathways. The top 10 biological processes of hypermethylated mRNAs (**a**) and hypomethylated mRNAs (**b**) in the *E. coli* group. The top 10 enriched pathways of hypermethylated mRNAs (**c**) and hypomethylated mRNAs (**d**) in the *E. coli* group.

In order to demonstrate the important function of m^6^A modification in MAC-T cells induced with *E. coli*, GO and KEGG enrichment analyses were performed on the above molecules with differential m^6^A methylation. Regarding the biological process function (BP) of GO enrichment analysis, we observed that genes with m^6^A hypermethylation in the *E. coli* group were mainly enriched in processes related to the regulation of signal transduction, protein modification, cell differentiation, etc. (Figure 4a), whereas genes with m^6^A hypomethylation were mainly enriched in RNA biosynthetic and cellular macromolecule biosynthetic processes (Figure 4b).

Furthermore, KEGG analysis annotated m^6^A hypermethylated genes in the *E. coli* group as participating in the MAPK signaling pathway, circadian rhythm, Ras signaling pathway, nitrogen metabolism, sphingolipid signaling pathway, etc. (Figure 4c), whereas m^6^A hypomethylated genes were significantly associated with the sulfur relay system, ribosome biogenesis in eukaryotes, the Wnt signaling pathway, the NF-κB signaling pathway, the Hippo signaling pathway, etc. (Figure 4d).

### 2.5. Conjoint Analysis of mRNA-Seq and MeRIP-Seq

**Figure 5:**
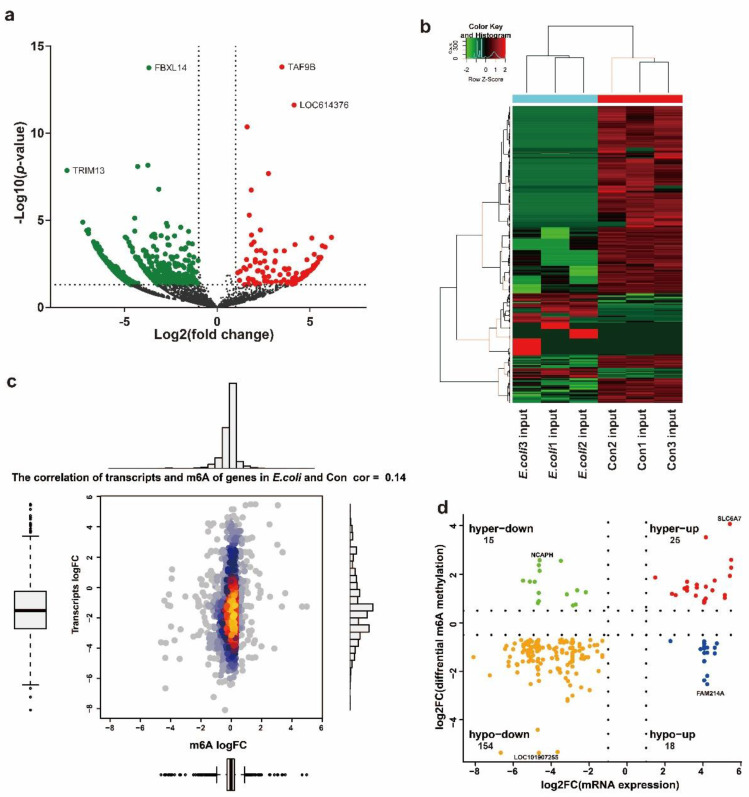
Conjoint analysis of m^6^A methylation and mRNA expression. (**a**) Volcano plots showing the differentially expressed mRNAs between the *E. coli* and Con groups with statistical significance (fold change ≥2.0 and *p <* 0.05). (**b**) Heat map demonstrating the differentially expressed mRNAs in three *E. coli* input samples and three control input samples. (**c**) Correlation of expression and m^6^A modification of transcripts in the *E. coli* group compared with the Con group. (**d**) Four-quadrant graph displaying the distribution of differential transcripts with m^6^A methylation and expression in the *E. coli* group.

To further explore the relationship between m^6^A modification and mRNA expression, we used RNA sequencing data from the input samples to analyze the mRNA expression of the control and *E. coli* groups. Volcano plots showed significantly different mRNA expression distribution in the *E. coli* group (*p* < 0.05, fold change >2.00) (Figure 5a). Hierarchical clustering showed the similarity of relative expression levels between the Con and *E. coli* groups (Figure 5b). On the basis of this analysis, we identified 190 upregulated and 595 downregulated genes in the *E. coli* group. Table 2 shows the top 20 most significantly different genes in the Con and *E. coli* groups.

A combined analysis showed differential mRNA expression levels (*p* <0.05, fold change >1.5) and m^6^A methylation peaks (*p* < 0.00001, fold change >1.5). The correlation coefficient between m^6^A modification and mRNA expression in the *E. coli* group compared with the Con group was 0.14 (Figure 5c), indicating a weak connection between m^6^A and the overall mRNA expression level. In addition, these genes were divided into four parts, including 25 hypermethylated and upregulated genes (hyper-up), 154 hypomethylated and downregulated genes (hypo-down), 15 hypermethylated but downregulated genes (hyper-down), and 18 hypomethylated but upregulated genes (hypo-up) (Figure 5d). Table 3 shows the 20 genes with significantly differential m^6^A modification (*p* < 0.00001, fold change >1.5) and mRNA expression (*p* < 0.05, fold change >1.5) in the Con and *E. coli* groups.

## 3. Discussion

Bovine MECs are the first barrier against invading microorganisms in the mammary gland. The pathogen recognition receptors (PPRs) on the cell surface activate pathogen-associated molecular patterns (PAMPs) after recognizing related pathogens. This major interaction initiates a series of downstream regulatory expression, leading to the expression and release of antimicrobial molecules, chemokines, and cytokines by host cells, even causing apoptosis [30]. Despite the volume of studies that exist on mastitis, there are still uncertainties about the pathogenesis of mastitis caused by *E. coli*. Therefore, the prevention, diagnosis, and treatment of mastitis remain challenging. The abnormality of m^6^A-modified enzymes can cause a series of diseases [31]; however, the mechanism of m^6^A modification in bovine mastitis remains unclear. This study is the first to analyze the relationship between the m^6^A modification profile and *E. coli*-induced mastitis.

Using MeRIP-seq to assess m^6^A modification in a mastitis model, we obtained an overview of m^6^A modification in mastitis treated with inactivated *E. coli* infection. The total m^6^A modification peak numbers revealed significant differences in m^6^A modification between the control and *E. coli* groups. Therefore, we assume that m^6^A modification may be related to *E. coli*-induced mastitis.

It is known that m^6^A modification of mRNA often affects changes in and the development of diseases. In this study, we identified a total of 2904 distinct m^6^A methylation peaks in 2101 mRNAs; 474 mRNAs had 622 hypermethylated peaks and 1627 mRNAs had 2260 hypomethylated peaks. Our analysis of the differential methylation patterns revealed some key mRNAs that may be closely related to mastitis (Figure 3a). BCL2 was found to be hypomethylated. As one of the key factors in the study of apoptosis, BCL2 can inhibit cell apoptosis and protect tissue [32,33]. Therefore, we suspect that m^6^A modification of BCL2 is involved in the process of apoptosis in mastitis. TLR4 is an important membrane protein receptor that can recognize lipopolysaccharides on the surface of Gram-positive bacteria such as *E. coli* [10,34,35], and TIRAP, a Toll/IL-1 receptor (TIR) domain of cohesion in cells, upon identifying PAMPs, can activate a series of downstream immune responses [36,37]. We speculate that the m^6^A hypomethylation in these two key molecules (TLR4 and TIRAP) by MeRIP-seq and IGV visualization reduces the rapid inflammatory reaction of MAC-T treated with *E. coli.* The findings regarding m^6^A modification of these genes suggest that differential m^6^A modification somewhat enriches the pathogenic mechanism of *E. coli* infection of bovine mammary glands. Although previous studies have confirmed the important role of some genes in the pathophysiology of mastitis, few studies have been conducted on m^6^A modification. Whether the changes resulting from the m^6^A modification of genes play an important role in mastitis caused by *E. coli* requires further verification.

*E. coli* can not only activate the natural immunity of dairy cow mammary gland tissues through the nucleotide binding oligomerization domain (NOD)-like receptor signaling pathway, the TLR signaling pathway, and the NF-κB signaling pathway, but it can also cause cell apoptosis [3,4,8]. Studies have confirmed that the Ras–MAPK signaling pathway affects proliferation, differentiation, and apoptosis by affecting gene transcription and regulation [38,39]. The NF-κB signaling pathway is a key regulatory pathway for immune response, apoptosis, and differentiation [40]. In our study, pathway enrichment analysis showed that the m^6^A differential methylation peaks were mainly enriched in the MAPK signaling pathway, NF-κB signaling pathway, Ras signaling pathway, and Hippo signaling pathway. This evidence also indicates that m^6^A modification is probably associated with mastitis.

Nevertheless, in the combined analysis of mRNA-seq and MeRIP-seq, we found a weak correlation [41] of m^6^A modification and mRNA expression between the *E. coli* group and the control group. Meanwhile, there were 212 genes in the *E. coli* group that exhibited differential methylation with significant differential expression of mRNA compared with the Con group (Figure 5a,b). The change in expression of these genes may be closely related to m^6^A modification [42]. These 212 genes (Table 3) included BAD, a protein in the BCL-2 family which plays a key role in mitochondrial-dependent apoptosis and participates in the development of many diseases by regulating cell death [43,44,45]. TMEM214 was also included, which acts as an anchor for the recruitment of procaspase 4 to the endoplasmic reticulum and its subsequent activation, and which is essential for endoplasmic reticulum stress-induced apoptosis [46]. MAP3K2, MAP2K1, and MAPK12 were also identified. Belonging to the MAPK cascade family of molecules, these proteins participate in important biological processes such as cell proliferation, differentiation, and immune response [34,35]. Whether the difference in m^6^A modification of these molecules affected the changes in their mRNA levels and, in turn, affected the release of inflammatory factors and the occurrence of cell apoptosis requires further experimental confirmation. In addition to affecting mRNA expression, m^6^A modification has many other functions, such as affecting the splicing of mRNA precursors, regulating the nuclear export of RNA, regulating mRNA translation, and affecting the stability of mRNAs [47,48]. The genes that did not exhibit expression changes may have one or more of these functions, which should be confirmed by further experiments.

**Figure 6:**
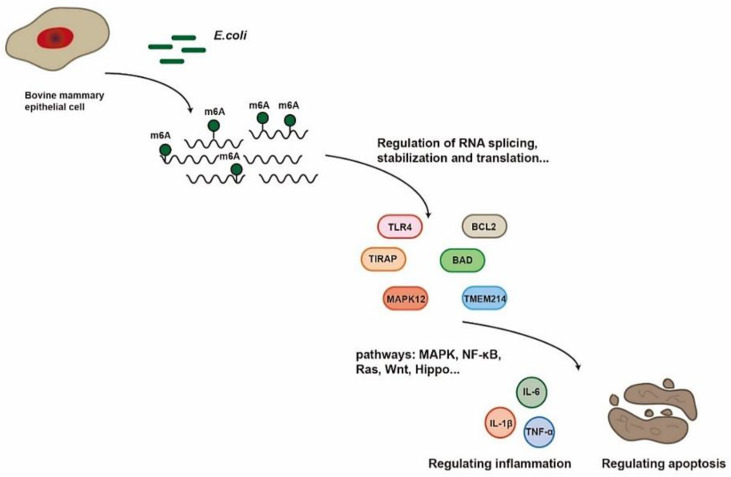
Sketch showing the potential pathways for MAC-T cells treated with the *E. coli*.

The model established using inactivated bacteria retained the main infectious components of *E. coli*, while avoiding bacterial growth and apoptosis. Inactivated bacterial infection model experiments have been widely recognized and applied in many mastitis studies [49,50,51]. Although MAC-T cells cannot fully represent the microenvironment during the natural infection process of mastitis, MECs are the first barrier in mastitis, not macrophages or neutrophils [30,51,52]. Therefore, MECs are suitable in vitro models for mastitis. We suspect that m^6^A modification affects the transcription and translation of mRNA in MAC-T cells treated with *E. coli* and, in turn, affects physiological and pathological processes such as inflammatory release and cell apoptosis (Figure 6). However, the mechanism of m^6^A-regulated mastitis in dairy cows is not clearly understood. Through the use of advanced technologies, we provide the first m^6^A transcriptome profile of mastitis and an initial map revealing the function of m^6^A modification in mastitis, thereby contributing critical insights for further research on the role of m^6^A in mastitis.

## 4. Materials and Methods

### 4.1. Bacteria Strains and Cell Line

*E. coli* (ATCC 25922) was donated by associated Professor Wang Xiangru of Huazhong Agricultural University, respectively. *E. coli* was resuscitated in LB (Luria–Bertani) solid medium. Single colonies were grown at 37 °C overnight. A single bacteria colony was used to inoculate culture bottle containing 10 mL LB broth and incubated in a shaker for 220 r/min. After counting the bacteria, the bacteria were heat-inactivated at 63 °C for 30 min.

MAC-T cells (an immortalized bovine mammary epithelial cell line) were donated by Professor Mark Hanigan of Virginia Tech University. The culture medium formulations of MAC-T cell differed from those reported in the literature [10]. MAC-T cells were cultured in DME/F12 medium (Hyclone, Tauranga, New Zealand) supplemented with 10% fetal bovine serum (Gibco, New York, NY, USA), and incubated at 37 °C in a 5% CO_2_ humidified incubator. The cells were digested and passaged using 0.25% trypsin and 0.02% EDTA.

### 4.2. Sample Collection and RNA Extraction

Trypsinized MAC-T cells were counted and seeded in a cell culture dish (Corning, New York, NY, USA) with 10^6^ cells per well. After 12 h, the DME/F12 maintenance medium containing 2% FBS was replaced. Three groups of cells were set up in triplicate. To the control group (Con) was added only the LB broth. The *E. coli* group was induced with 10^7^ inactivated *E. coli* at a multiplicity of infection (MOI) of 10:1 and incubated for 24 h. The medium was discarded, and the cells were gently washed thrice using cold PBS (Hyclone, Tauranga, New Zealand), before adding 1 mL of TRIzol reagent (Invitrogen, Carlsbad, CA, USA) to each well for RNA cell lysate collection.

Total RNA was extracted according to the commercial reagent manufacturer’s instructions (Invitrogen) and RNA isolation procedures. The NanoDrop 2000 instrument (Thermo Fischer Scientific, Waltham, MA, USA) was used to measure the RNA concentrations, and the RNA purity index was denoted by an OD_260_/OD_280_ value between 1.80 and 2.10. The integrity of the sample RNA and potential gDNA contamination were assessed using agarose gel electrophoresis.

### 4.3. Real-Time Quantitative PCR

HiScript reverse transcriptase (Vazyme, Nanjing, China) was used to reverse-transcribe the RNA samples. The corresponding mixture of cDNA was configured according to AceQ SYBR^®^ qPCR Master Mix (Vazyme), and the expression levels of IL-1β, IL-6, and TNF-α were detected using a ViiA7 Real-Time PCR System (Applied Biosystems Inc., Foster, CA, USA) instrument. The 2^−ΔΔCt^ method was used for data analysis. Related primer sequences were as follows: β-actin F, AGATCAAGATCATCGCGCCC and R, TAACGCAGCTAACAGTCCGC; IL-1β F, TTCCATATTCCTCTTGGGGTAGA and R, AAATGAACCGAGAAGTGGTGTT; IL-6 F, CAGCAGGTCAGTGTTTGTGG and R, CTGGGTTCAATCAGGCGAT; TNF-α F, TCTTCTCAAGCCTCAAGTAACAAGC and R, CCATGAGGGCATTGGCATAC.

### 4.4. Enzyme-Linked Immunosorbent Assay (ELISA)

Cell-free supernatants were collected from the groups of bacteria treatments and assayed for proinflammatory cytokines (IL-1β, IL-6, TNF-α) using an ELISA kit (Cusabio, Wuhan, China). Based on the manufacturer’s instructions, the OD of each sample was assessed with a microplate reader at 450 nm wavelength. A standard curve was constructed to calculate the concentration of the samples.

### 4.5. Flow Cytometry

MAC-T cells were digested with trypsin without EDTA for 3 min. After stopping the digestion with DMEM/F12 containing 10% FBS, the cells were collected by centrifugation. After washing three times with PBS, the cells were re-suspended with 100 μL of Binding Buffer. Then, 5 μL each of FITC and PI dyes (Vazyme) were added and left for 10 min at room temperature in the dark. After adding 400 μL of Binding Buffer, the cells were detected using cytoflex-LX (Beckman Coulter, Indianapolis, IN, USA).

### 4.6. Methylated RNA Immunoprecipitation (MeRIP)

Firstly, the collected RNA was fragmented. Secondly, protein A magnetic beads (Thermo Fisher Scientific, Waltham, MA, USA) were incubated with anti-m^6^A antibody (abcam, Cambridge, UK) at room temperature for 1 h. Thirdly, the recovered RNA fragments were co-incubated with the mixture of magnetic beads and antibodies at 4 °C for 3 h. Fourthly, the above mixture was eluted with elution buffer, and the collected RNA was reverse-transcribed by HiScript reverse transcriptase. Lastly, the corresponding mixture of cDNA was configured according to AceQ SYBR^®^ qPCR Master Mix (Vazyme), and the expression levels of BCL2 and TLR4 were detected using a ViiA7 Real-Time PCR System instrument. The percentage input method was used for data analysis, where % input = 2^−(Average CTRIP − AverageCTinput − log2(input dilution factor))^. Related primer sequences were as follows: TLR4 F, CCGGCTGGTTTTGGGAGAAT and R, ATGGTCAGGTTGCACAGTCC; BCL2 F, CAGTTGCTCTGCTGTTTGAGG and R, CATTACTCTAGTGCTCCCCGC.

### 4.7. MeRIP-seq and mRNA-seq

The extracted RNA samples were sent to Cloud-Seq Biotech (Shanghai, China) for MeRIP-seq and mRNA-seq analyses (GSE 161050). Firstly, the fragmented RNA and m^6^A antibodies (Millipore, Burlington, MA, USA) were incubated in IP buffer at 4 °C for 2 h. Secondly, the mixture was immunoprecipitated with protein A magnetic beads (Thermo Fisher Scientific, Waltham, MA, USA) at 4 °C for 2 h. Thirdly, the RNA on the magnetic beads was eluted with a free m^6^A adenosine analogue and further extracted with TRIzol reagent. Lastly, the m^6^A IP samples and the input samples without immunoprecipitation were used for the NEBNext^®^ Ultra II Directional RNA Library Prep Kit (New England Biolabs, Inc., Ipswich, MA, USA), and double-ended sequencing was performed on an Illumina Hiseq (Illumina, Inc., San Diego, CA, USA).

### 4.8. Bioinformatics Analysis

Image analysis, base recognition, and quality control were performed after sequencing to produce raw data of original reads. Next, Q30 quality control and Cutadapt software (V1.9.3) (http://code.google.com/p/cutadapt/, accessed on 25 March 2020) were used to remove the connector and low-quality reads, thereby obtaining high-quality clean reads (Table S1, Supplementary Materials). The reads were compared on the basis of the genome/transcriptome (bosTau9) using Hisat2 software (v2.0.4) [53], and the MACS software (v1.4.2) [54] was used to identify methylated mRNA peaks in each sample. IGV software (v2.4.10) [55] was used to visualize the matching of MeRIP and input on the genome. The Gene Ontology (GO) (http://www.geneontology.org, accessed on 27 April 2020) and Kyoto Encyclopedia of Genes and Genomes (KEGG) (http://www.genome.jp/keg, accessed on 27 April 2020) databases were used to analyze differentially methylated genes.

### 4.9. Statistical Analysis

Data analysis was performed using GraphPad Prism v7.0 software, and values are reported as the mean ± standard deviation (SD) of at least three replicates in each group. A *p*-value of <0.05 was considered statistically significant (*, **, and *** represent *p* < 0.05, *p* < 0.01, and *p* < 0.001, respectively).

## 5. Conclusions

Our findings clearly profile the m^6^A methylation of *E. coli*-induced MAC-T cells and identify many differentially methylated genes, which may contribute to the diagnosis and treatment of mastitis. These results are the first to elucidate a potentially meaningful relationship between m^6^A modification and dairy cow mastitis, laying the foundation for future studies of m^6^A modification in mastitis.

## Figures and Tables

**Table 1:** The top 20 differently expresses m^6^A peaks between *E. coli* and controls based on *p*-value.

Gene Name	Peak Region	Peak Start	Peak End	Chromosome	Log10 (*p*-Value)	Log2 (Fold Change)	Regulation
TOR1AIP1	startC	61,095,201	61,095,890	NC_037343.1	−9.64	1.09	hyper
SYTL2	CDS	9,831,641	9,831,860	NC_037356.1	−9.42	1.21	hyper
HERC6	startC	36,366,461	36,366,789	NC_037333.1	−8.98	1.44	hyper
BHLHE41	startC	83,818,001	83,818,465	NC_037332.1	−8.9	2.57	hyper
GPR155	CDS	22,358,286	22,358,700	NC_037329.1	−8.84	2.45	hyper
IGFBP5	stopC	104,660,241	104,660,804	NC_037329.1	−8.82	1.44	hyper
TSPAN18	3′UTR	75,001,041	75,002,400	NC_037342.1	−8.68	1.74	hyper
CENPU	3′UTR	15,119,101	15,119,520	NC_037354.1	−8.64	1.73	hyper
AKAP13	CDS	16,514,463	16,514,712	NC_037348.1	−8.6	1.08	hyper
RFX2	3′UTR	18,396,043	18,397,660	NC_037334.1	−8.58	1.03	hyper
CLCA2	CDS	57,619,160	57,619,431	NC_037330.1	−10.75	7.39	hypo
NUDT19	3′UTR	43,175,361	43,175,740	NC_037345.1	−10.52	4.43	hypo
FZD2	CDS	44,329,981	44,330,600	NC_037346.1	−10.50	4.07	hypo
LOC101907255	5′UTR	41,829,801	41,830,640	NC_037353.1	−10.32	7.74	hypo
LOC101903326	CDS	1,089,721	1,090,900	NC_037341.1	−10.32	5.05	hypo
HGFAC	CDS	115,444,475	115,444,760	NC_037333.1	−10.27	2.78	hypo
GPR156	CDS	65,018,452	65,018,905	NC_037328.1	−10.11	3.88	hypo
AOAH	CDS	60,547,451	60,547,800	NC_037331.1	−10.11	3.33	hypo
AAR2	3′UTR	65,379,401	65,379,780	NC_037340.1	−10.09	3.98	hypo
STRBP	3′UTR	94,269,361	94,269,860	NC_037338.1	−10.09	4.54	hypo

**Table 2:** Top 20 differential mRNA expression in *E. coli* vs. con.

Gene Name	Log2 (Fold Change)	Log10 (*p*-Value)	Regulation
IER3	6.164040553	−4.02169998	up
PTGS2	5.826757014	−3.467281725	up
ERO1A	5.669337971	−3.536666229	up
PRDX5	5.586646738	−2.881586777	up
HNRNPC	5.556401381	−2.802666029	up
CA4	5.481535598	−2.86568667	up
SLC6A7	5.414589103	−2.589570251	up
TMCC3	5.325086008	−2.488894255	up
DDIT4	5.280240307	−2.400378604	up
SLC2A1	5.237809067	−3.050012716	up
TRIM13	−8.101651804	−7.866625717	down
CD70	−7.242189071	−4.903814594	down
DDX39A	−7.027795258	−4.414552565	down
YEATS2	−6.971898132	−4.337112177	down
RASGRP1	−6.946317893	−4.286252914	down
PRPF38A	−6.946269946	−4.239872373	down
CELSR3	−6.935875444	−4.464239943	down
AQP11	−6.664329625	−3.773669927	down
GAPDHS	−6.638835912	−3.724064388	down
LOC112442013	−6.591469341	−3.591651156	down

**Table 3:** All 20 transcripts of differential m^6^A modification and mRNA expression in *E. coli* vs. Con.

Gene Name	Change	Chromosome	m^6^A Modification Change						mRNA Expression Change		
			Peak Start	Peak End	Peak Length	Peak Region	logFC	Log10 (*p*-Value)	logFC	*p*-Value	Strand
MAP3K2	hyper-up	NC_037329.1	5,150,437	5,150,820	383	5′UTR	2.49	−6.97	4.071	0.021139	+
ECI1	hyper-up	NC_037352.1	1,765,100	1,765,420	320	CDS	2.33	−5.30	4.07	0.023957	+
ALK	hyper-up	NC_037338.1	70,660,381	70,661,760	1379	CDS	1.98	−6.28	3.11	0.002504	−
CA4	hyper-up	NC_037346.1	12,807,579	12,807,920	341	CDS	13.42	−7.90	5.48	0.001362	+
SEMA6B	hyper-up	NC_037334.1	19,583,616	19,585,160	1544	CDS	3.81	−6.16	4.49	0.022398	+
HK1	hyper-down	NC_037355.1	25,767,278	25,767,520	242	CDS	1.84	−5.07	−4.66	0.047763	+
GAB2	hyper-down	NC_037356.1	17,787,967	17,788,070	103	3′UTR	1.85	−5.68	−2.71	0.042939	+
MAP2K1	hyper-down	NC_037337.1	13,276,353	13,276,712	359	5′UTR	3.52	−5.06	−2.18	0.023033	+
CTSB	hyper-down	NC_037335.1	7,566,076	7,566,228	152	5′UTR	1.73	−5.81	−5.22	0.015551	+
EIF2AK4	hyper-down	NC_037337.1	35,645,637	35,645,717	80	CDS	1.69	−5.17	−3.56	0.00288	+
USP1	hypo-down	NC_037330.1	83,118,461	83,118,920	459	3′UTR	1.60	−6.82	−1.67	0.023734	−
DDX58	hypo-down	NC_037335.1	11,604,221	11,604,700	479	3′UTR	3.30	−8.74	−5.00	0.021294	+
FBN1	hypo-down	NC_037337.1	61,917,881	61,918,380	499	CDS	4.14	−7.63	−2.17	0.001185	+
BAD	hypo-down	NC_037356.1	42,573,697	42,574,197	500	3′UTR	1.56	−5.22	−4.35	0.045042	−
MAPK12	hypo-down	NC_037332.1	119,619,701	119,620,440	739	3′UTR	6.15	−6.27	−4.73	0.043358	−
TMEM214	hypo-up	NC_037338.1	72,593,801	72,594,100	299	3′UTR	1.60	−7.89	4.14	0.042666	−
STAT2	hypo-up	NC_037332.1	57,007,910	57,008,239	329	CDS	2.11	−7.11	2.28	0.046055	+
ACOX3	hypo-up	NC_037333.1	114,662,000	114,662,840	840	CDS	2.14	−5.17	4.06	0.045482	−
RASSF6	hypo-up	NC_037333.1	88,624,201	88,624,540	339	3′UTR	2.79	−7.76	4.61	0.017354	−
ZNF385A	hypo-up	NC_037332.1	25,706,604	25,707,959	1355	CDS	2.87	−6.82	4.06	0.034321	+

## Data Availability

https://www.ncbi.nlm.nih.gov/geo/query/acc.cgi?acc=GSE161050, accessed on 9 November 2020.

## Supplementary Materials

The following are available online at https://www.mdpi.com/article/10.3390/ijms22126254/s1, Figure S1: Overview of the m6A methylation map in the Con and *E. coli* groups, Table S1:The detailed information of the raw data, Table S2: Differentially methylated RNA sites for: *E. coli* vs. Con.
