# Supplementary figures and images for: Transcriptome Profiling of m6A mRNA Modification in Bovine Mammary Epithelial Cells Treated with Escherichia coli
Source: Int J Mol Sci. 2021 Jun 10;22(12):6254. doi: 10.3390/ijms22126254 (PMC8230414; doi:10.3390/ijms22126254)

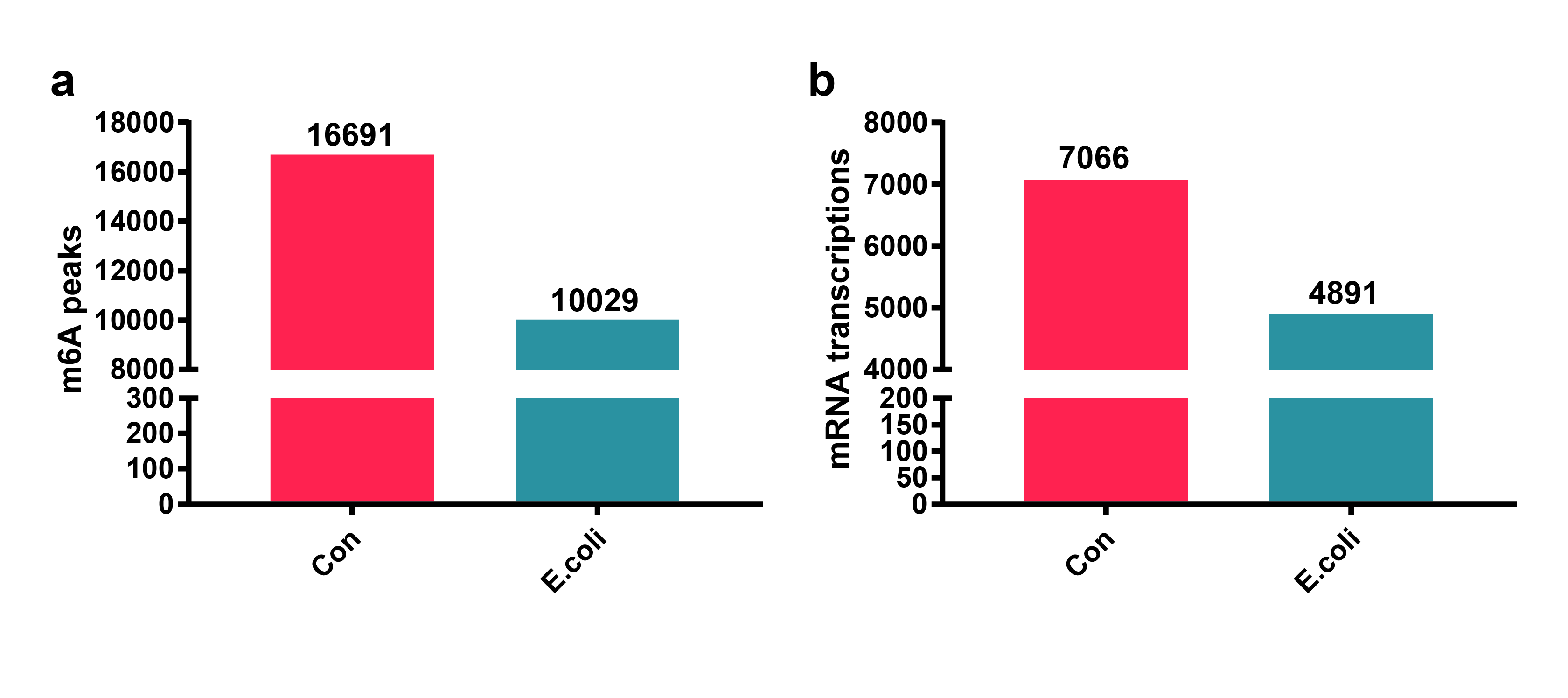

Supplement: Supplementary file 1 [file ijms-22-06254-s001.zip › supplementary files/Figure S1.tif]
